# Social identity switching: An investigation of non‐demographic identities with computational‐linguistic and self‐report measures

**DOI:** 10.1111/bjso.12906

**Published:** 2025-05-21

**Authors:** Anna Kristina Zinn, Aureliu Lavric, Elahe Naserianhanzaei, Miriam Koschate

**Affiliations:** ^1^ Department of Psychology University of Exeter Exeter UK; ^2^ UQ Business School The University of Queensland Brisbane Queensland Australia; ^3^ Department of Political Sciences University of Exeter Exeter UK; ^4^ Institute for Data Science and AI University of Exeter Exeter UK

**Keywords:** identity activation costs, linguistic style, multiple identities, social identity, social identity switching

## Abstract

Understanding potential costs of social identity switching contributes to our knowledge of how people manage their various group memberships in a fast‐paced and interconnected world. Recent research demonstrates that people can switch between demographic social identities seamlessly. The current studies advance this research by (1) moving beyond demographic identities to identities that are not perceptually distinguishable, (2) developing a new identity switching paradigm based on an implicit computational linguistic style measure of salience and (3) including self‐report measures of salience, task difficulty and performance. In two within‐subjects studies (*N* = 211; *N* = 220), a short writing task was used to prompt a switch from participants' parent identity to their feminist identity or a repetition of the feminist identity. Findings from the implicit measure revealed no identity activation ‘cost’ in the switch relative to the repeat condition, consistent with previous findings for demographic identities. In contrast, we found evidence for lower self‐reported salience of the feminist identity in the switch compared to the repeat condition. Furthermore, Study 2 found little difference in self‐rated performance or task difficulty between conditions, indicating that switching identities does not affect self‐rated performance. The results illustrate a new paradigm for investigating social identity switching.

## INTRODUCTION

Being able to switch seamlessly between our many different social identities enables context‐appropriate behaviour that is in line with group norms and values (Oakes, 1987; Turner et al., 1987). For instance, in an everyday scenario, a person might quickly reply to a message from their child and then return to typing an important work‐related email. A seamless switch in this scenario would ensure a high correspondence between the person's behaviour and the goals and norms of each identity, resulting in a caring response in casual language to their child and then continuation of the work email in a suitably professional tone.

Recent research shows that switches between demographic social identities, such as ethnic group and age category, can be very effective—causing no significant delay in, or weakening of, the activation of the switched‐to identity (Zinn et al., 2022). Here, we test whether switches between non‐demographic social identities that are not visually distinguishable—such as identities based on collective action (e.g. feminist) or relationship roles (e.g. parent)—are seamless, or whether a switch inhibits the activation of the next identity, even if only for a short moment. To do so, we introduce a paradigm that uses a computational linguistic style measure of social identity salience. We further aim to expand our knowledge on social identity switching by examining its effects on the subjective experience of switching as well as on self‐reported performance and task difficulty for the first time.

### Effectiveness of social identity switching

Over 40 years of social identity research has documented the important role social identities play in people's everyday lives. An individual's social identity is derived from the various groups and category memberships they identify with (Tajfel & Turner, 1979). The wide range of groups and categories can be based on demographics such as one's gender identity, personal relationships, such as being a parent, organizational memberships, such as being an employee in a particular company or shared values and opinions, such as being a feminist (e.g. Cork et al., 2023; Deaux et al., 1995; Lickel et al., 2000; Prentice et al., 1994). The different social identities have in common that they are a key part of the individual's sense of self and that they can shape one's thoughts, perceptions, emotions and behaviour as a result (Tajfel & Turner, 1979; Turner et al., 1994).

Importantly, our social identities do not affect us all simultaneously. Instead, the social context makes the most relevant social identity salient, that is, it activates it in our mind along with the norms and values associated with that group or category (Oakes, 1987; Turner et al., 1994). For example, when exiting the bus and walking into the hospital, a doctor might switch from a customer identity to their work‐related identity. While in the hospital, they might receive a text message from their child, eliciting a switch to their parent identity. According to self‐categorization theory (SCT), such social identity switches occur in a natural and seamless way (e.g. Turner et al., 1994).

To empirically test whether we can, indeed, move seamlessly between social identities, a paradigm was developed by Zinn et al. (2022) based on the so‐called task‐switching paradigm (e.g. Kiesel et al., 2010; Meiran, 1996; Monsell, 2003, 2015; Rogers & Monsell, 1995). Task‐switching studies directly compare performance during task switches vs. task repetitions. A participant might, for instance, either switch between identifying the colour of an object and identifying its shape or continue to identify the colour (e.g. Lavric et al., 2008; Monsell & Mizon, 2006). This comparison revealed task ‘switch costs’: longer response times and higher error rates on task switch trials compared with task repeat trials (Rogers & Monsell, 1995). For instance, participants take longer to complete trials and make more mistakes when they switch between identifying colours and shapes compared to continuing to identify colours.

Building on the background of task switching research, Zinn et al.'s (2022) studies directly compare social identity switches and repetitions in a within‐subject design. In task switching research, this comparison focuses on immediate performance measures such as reaction time and accuracy. Instead, for identity switching, the focus is on *identity activation costs*, that is, ‘a delayed or weaker activation of the switched‐to identity’ (Zinn et al., 2022, p. 1). In recent studies on switching effectiveness, identity salience was derived from an identity‐based Implicit Association Test (IAT; Greenwald et al., 1998; Pinter & Greenwald, 2010; see Zinn et al., 2022, for further discussion of similarities and differences between the task‐switching and identity‐switching paradigms and information on the IAT as a salience measure). Studies using this paradigm have so far revealed no significant differences in identity salience between identity switches and identity repetitions—and hence no detectable identity activation costs (Zinn et al., 2022). In the first study, this was shown for switches between well‐established demographic identities (age and race). The second study showed similar results for switches between well‐established demographic groups (nationality and age) and for switches from a newly acquired minimal group (red vs. blue group) to a well‐established demographic group (age). While this research on social identity switching provided initial evidence that social identity switches are effective, key knowledge gaps remain. In what follows, we outline these gaps and how the present study aims to address them.

### Social identity switching between non‐demographic groups

Social identities derive from many different kinds of groups and categories, with demographic categories forming only one of several types of group memberships (Cork et al., 2023; Deaux et al., 1995; Lickel et al., 2000). As Zinn et al.'s (2022) research focused predominantly on demographic groups, it remains unclear whether switches between other types of identity are also effective. For instance, according to ratings obtained by Lickel et al. (2000), demographic (‘social’) categories are lower in entitativity, personal importance and levels of member interactions than ‘intimacy’ and ‘task‐based’ groups but higher in durability and size. Cork et al. (2023) found that demographic groups are higher in conformity, universalism and self‐direction compared to ‘(a)vocational’, ‘relationship’ and ‘stigmatized’ groups that were defined more by achievement and benevolence, respectively. Thus, Zinn and colleagues' findings may not hold true for switches between non‐demographic groups. The first aim of the current study is, therefore, to examine the effectiveness of identity switching for non‐demographic groups.

#### Implicit assessment of salience for groups that do not differ in physical appearance

So far, identity activation costs have been assessed with implicit measures that rely on visually distinguishable group characteristics (Zinn et al., 2022), such as the IAT (Greenwald et al., 1998) and the identification IAT (Pinter & Greenwald, 2010). However, such measures are harder to apply to social identities that cannot be distinguished easily by physical appearance, such as identities based on shared opinions (e.g. feminist) or on relationship roles (e.g. parent). Zinn et al. (2022) tried to address this challenge for minimal groups by requiring participants to learn the faces of in‐group members prior to the IATs. However, this procedure substantially lengthened the experimental session and would make it even longer and more difficult if in‐group and out‐group membership had to be learned for each face for not only one but also two different categories.

A fundamentally different implicit measure, which was explicitly created to assess social identity salience and does not rely on visually distinguishable characteristics, is the Automated Social Identity Assessment (ASIA; Koschate et al., 2021). The basic premise of ASIA is that the style in which we write in a particular moment is affected by the norms and values of the social identity that is salient. For instance, when our academic identity is salient, we are likely to use an ‘academic style’ that is intellectual, concise and unemotional, irrespective of the topic we are writing about. In contrast, when we write as a friend, we are more likely to use an informal and emotive style, even if we write about the same topic and to the same person but in different social contexts (e.g. telling an academic who is a friend about an exciting finding in a personal email versus responding to them as a reviewer of a manuscript). This change of style shows that we use social identities flexibly even when we highly identify with both categories, for example, as an academic and as a friend.

ASIA assesses the *relative salience* between two social identities, such as between a feminist and a parent identity, by using stylistic elements of writing (Koschate et al., 2021). Stylistic elements include the length of words, grammar (e.g. number of pronouns), punctuation and general word categories (e.g. emotion words, time words). As a result, ASIA is not reliant on participants talking about their identity or using identity‐related words (e.g. feminist, women; parent, children) or vernacular associated with a particular identity (e.g. objectification; tummy). For instance, the words ‘woman’ or ‘child’ are simply counted as a noun, thereby stripping the content information from the word. This makes ASIA less prone to false classification (e.g. non‐parents talking about children) or outright manipulation and flexible regarding the topic. A pre‐trained and validated ASIA model can be used to provide probability scores that indicate which of the two identities was more likely to be salient when the participant wrote the text.

Koschate et al. (2021) and Cork et al. (2020) demonstrated that a trained classifier is able to determine relative identity salience (e.g. feminist vs. parent; libertarian vs. entrepreneur) with a relatively high accuracy of 72–75% correct classifications. The classifier retained its accuracy in an experimental design in which participants who held both identities were randomly assigned to conditions in which only one of the two identities was made salient. Importantly, the writing topics and the audience were kept constant over conditions in this experimental validation.

The parent‐feminist ASIA model has already been successfully used to measure identity salience in an experiment that examined whether participants could intentionally prevent an identity switch (Zinn et al., 2023). Participants in the experimental condition were asked to stay in their parent identity and were incentivized through additional payment. In contrast, the control condition was not told which identity should be salient. Self‐report measures indicated that participants thought they had remained in their parent identity as instructed in the experimental condition. The implicit salience measure, however, revealed an equivalent switch from the parent to the externally activated feminist identity in both conditions. Interestingly, the result was not affected by differences in the use of relevant content words: Participants in the experimental condition used more family words (e.g., daughter, son) than participants in the control condition.

Thus, ASIA allows the direct measurement of the relative salience of specific identities without relying on features such as physical appearance. Furthermore, once the classifier is trained, tested, and validated (prior to the experiment) for the identities of interest, the only requirement during the experiment is that participants write a piece of joined text (prose) of at least 25 words. Building on past research into ASIA, the second aim of our study is to test a new identity switching paradigm that uses ASIA to assess the effectiveness of social identity switches.

#### Subjective experience of social identity switches

Implicit measures may reveal endogenous processes into which people have limited insight/introspection. However, they do not capture how participants experience social identity switches. So far, research on social identity switching has only focused on implicit measures of identity switching (Zinn et al., 2022, 2023). To capture both the cognitive processes underlying social identity switching, as well as the experience of the switching process, implicit measures should be complemented by explicit self‐report measures of salience.

In task switching research, there has been interest in people's awareness of the task switch cost. The presence of such awareness is suggested by the finding that participants show a bias towards repeating the same task when given the opportunity to decide whether to switch or repeat a task (e.g., Mittelstädt et al., 2018). Research on the level of introspection has revealed a high correlation between self‐reported response times and actual response times in single‐task contexts (Corallo et al., 2008).

By extrapolation, one may expect that people's experience of identity switching might be closely aligned with the effectiveness assessed via implicit measures. However, this has not been tested thus far, except for the identity switch control study by Zinn et al. (2023), which provided initial evidence for a potential discrepancy between an implicitly measured and self‐reported social identity switch. As already mentioned, participants reported that they were still in a parent identity even though the implicit measure showed that they had switched to a feminist identity. However, this research focused on controlling identity switches rather than on the effectiveness of switches when participants are instructed to switch. Furthermore, participants may have been motivated to report that they were still in the parent identity as they expected to be paid extra if they managed to do so. Thus, the third aim of the present research is to include an explicit self‐report measure of identity salience for assessing the experienced effectiveness of switching identities.

#### Self‐reported performance and difficulty

Past research on social identity switching has focused exclusively on whether people incur identity activation costs—a delay of activating the switched‐to identity. However, a further key aspect is their experience of potential costs related to the task they are completing while switching identities. While the subjective experience of social identity switching—as discussed in the previous section—focuses on the experience of identity salience, this concept might be too abstract for participants to report on. In contrast, participants might be able to report more readily on how difficult they found a task or how they rated their performance on this task.

To further investigate the experience of social identity switching, the fourth aim of the present research is to investigate the effect of identity switching on self‐reported performance and difficulty. We will address this aim in our second study.

## CURRENT RESEARCH

The research by Zinn et al. (2022) discussed above provided the first empirical evidence that social identity switches are highly effective. The following two pre‐registered studies extend this research by attempting to (1) examine whether the key finding generalizes to social identities that are not based on demographic, perceptually distinguishable categories; (2) use a different implicit measure of identity salience and (3) additionally assess the subjective experience of switching effectiveness, performance and task difficulty.

The social identities of being a parent and being a feminist were chosen for the current study because members of these groups are not perceptually distinguishable, both identities relate to groups that are not based on demographics, they can be held independently of each other but can also be held by the same individual, and they are meaningful to a substantial number of people across the world, which allows us to include participants from different backgrounds (e.g. nationalities, ethnic groups). Although feminism is often associated with the female gender, people of other genders, including men, can identify as feminists (e.g. Silver et al., 2019). Similarly, parenting is often associated with the female gender due to the gendered nature of social roles in many societies, but people of other genders also strongly identify as parents. The choice of these two identities allows us to test the effectiveness of switching between two distinct groups of social identities (see, e.g. Cork et al., 2023): a ‘relationship’ identity (such as being a parent) and a ‘collective action’ identity (which includes the feminist identity, but also many other political as well as ethnic/religious identities). Furthermore, the choice of the parent and feminist identities allows us to take advantage of the fact that an ASIA classifier has been previously trained and extensively validated for these identities (Koschate et al., 2021).

The present studies consist of two experimental conditions—‘identity repeat’ and ‘identity switch’—both of which were completed by all participants, at least 1 week apart. Each condition included two writing tasks with the writing topic used as a prompt to make either the parent or feminist identity salient (see, e.g. Zinn et al., 2023). In the repeat condition, participants remained in the same identity. Hence, they wrote about a topic relevant to their feminist identity in both writing tasks. In the switch condition, participants were prompted to switch from a parent identity to a feminist identity. Hence, they were first asked to write about a topic relevant to their parent identity and then about a topic related to their feminist identity. Accordingly, the key difference between the switch and repeat condition was the social identity activated in the first writing task. This allowed for a direct comparison of identity switches and identity repeats, as in task switching research (e.g. Meiran, 1996; Rogers & Monsell, 1995) and as in the previous study on the effectiveness of social identity switching (Zinn et al., 2022).

### Hypotheses

If social identity switches are costly—leading to a delay in the activation of the subsequently prompted identity—a stronger feminist (rather than parent) linguistic style is expected in the feminist identity repeat condition as compared to the parent‐to‐feminist switch condition. This means that it should be more difficult for the parent‐feminist ASIA model to distinguish between the two identities after a switch than after a repeat of the same social identity. However, based on previous research (Zinn et al., 2022), in which no social identity activation costs were found, the following null hypothesis was tested for the implicit salience measure:

If social identity switches are effective, no significant difference in the implicit salience measure of the switched‐to identity is expected between the identity switch vs. repeat condition.

If switches are perceived as costly, higher self‐reported salience for the feminist identity in the repeat condition compared with the switch condition is expected. While there is no prior research on the level of introspection people have about social identity switches, we had no reason to believe that participants would perceive a switch as costly if the cognitive process is highly effective. Therefore, we did not expect to find perceived costs of identity switches, resulting in the second null hypothesis:

If social identity switches are perceived to be effective (no perceived identity activation cost), no significant difference is expected in self‐reported salience of the switched‐to identity between the identity switch vs. repeat condition.

Additionally, in Study 2, we tested whether social identity switching affected the self‐reported performance in the specific study task and difficulty ratings. Past findings and theory support that social identity switching is effective. If the relevant identity has been successfully activated, we would expect no costs on subsequent tasks. In other words, we would expect that the most suitable identity for the task has been activated. Therefore, we expect no effects of switching on task performance and difficulty perceptions. This resulted in the following null hypotheses.

If social identity switches are effective, no significant difference in the text performance ratings is expected between the identity switch and repeat condition.

If social identity switches are effective, no significant difference in the writing difficulty ratings is expected between the identity switch and repeat condition.

## STUDY 1

### Participants and design

Sample size calculations were based on Brysbaert (2019). We aimed to test for a statistical power of .80 for a small to medium effect size of Cohen's *d* = 0.40 in a within‐subject design. A paired‐samples t‐test requires 215 participants to test the null hypothesis under these requirements for H1 and H2.

Participants were recruited through the online Platform Prolific Academic. To take part in the study, they had to be a parent and consider themselves to be a feminist, be aged 18 or above, and have English as their mother tongue. Only participants who had not taken part in any of our studies on social identity switching on Prolific Academic and had completed at least five previous studies on the platform were invited to the study (the latter requirement ensured that participants used the recruitment platform regularly and were therefore likely to return to complete the second part of the study).

From the total of *N* = 258 participants who completed the first part of the study, eight did not meet the inclusion criteria (*n* = 2 were not parents; *n* = 6 did not list English as a mother tongue). This resulted in *N* = 250 participants being invited to the second part of the study. A total of *N* = 227 of them completed the second part of the study. However, *n* = 16 had to be excluded, with *n* = 15 for writing less than 25 words in one of the main texts, and *n* = 1 who did not indicate their first language. This resulted in a final sample size of *N* = 211 (153 women (72.5%), 57 men (27.0%), 1 non‐binary), which was close to our target of *N* = 215. Participants were, on average, 40.53 years old (*SD* = 10.04, Min = 23, Max = 65). All participants spoke English as one of their first languages, with *n* = 24 (11.4%) participants being bi‐ or multi‐lingual. Study 1 included participants from 12 different nationalities, with the majority of participants from the United Kingdom (57.3%), followed by South Africa (12.3%) and the United States of America (11.4%). Most participants (76.3%) indicated White as their race, followed by Black, Caribbean or African (14.2%), Asian or East Asian (4.3%) and Mixed or Multiple groups (3.8%), with three other or missing responses (1.5%; see Appendix A for a full list of nationalities and additional demographics).

The pre‐registered study has a within‐subjects design with switching (identity switch vs. repeat) as the independent variable and social identity salience as the dependent variable. Social identity salience was assessed both with an implicit measure (ASIA) and an explicit measure. A specific social identity was made salient by the topic participants were asked to write about. The order of the two feminist writing topics for the main writing task and whether participants started with an identity switch or repeat were counterbalanced. This resulted in four balancing conditions: switch and topic A first (*n* = 52); switch and topic B first (*n* = 55); repeat and topic A first (*n* = 52); repeat and topic B first (*n* = 52).^1^ The study received approval from the departmental ethics committee at the University of Exeter.

### Materials and procedure

**FIGURE 1:**
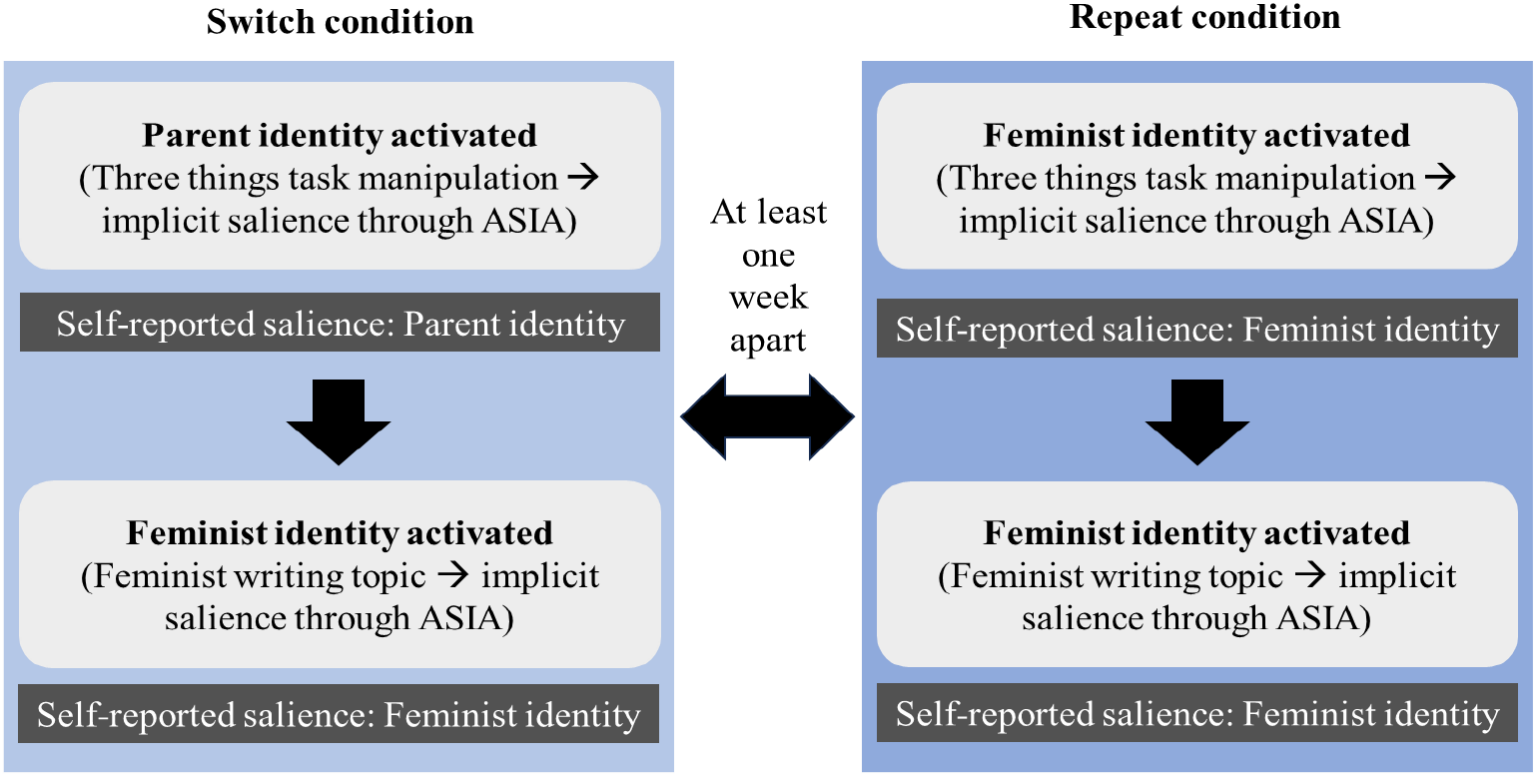
Study procedure. The order of the repeat and switch condition and that of the feminist writing topics were jointly counterbalanced over participants.

Participants provided informed consent to take part in the study, which was run online on Qualtrics. The study needed to be completed on a PC to avoid automatic sentence completion or notifications. Participants were told that the study consisted of two parts, each taking about 12 minutes, to be completed at least one week apart. They were paid £1.50 for the first and second parts of the study, respectively, plus a £1 bonus for completing both parts. Each participant completed the switch from the parent identity to the feminist identity and the repetition of the feminist identity in two separate Qualtrics surveys one week apart. Half of the participants started with the switch condition and the other half with the repeat condition. The main procedure of the study is summarized in Figure 1. Participants were either debriefed after the second part of the study or sent the debrief form via Prolific.

#### Salience manipulation of the first identity

The first writing task functioned as a social identity salience manipulation where either a parent identity in the switch condition or a feminist identity in the repeat condition was made salient. The analysis of the written answer served as a manipulation check. For this writing task, we adapted the three things manipulation by Haslam et al. (1999). Participants were asked to write at least 4–5 sentences (25 words) about the parent[/feminist] social identity by describing things that they and other parents[/feminists] do often, rarely, well and badly.

#### Salience of the second identity

The second writing task required participants in both conditions to write about one of two feminist topics (one topic per session): Topic A ‘Women are still treated like sex objects’. and Topic B ‘Women are still treated as the “weak gender” in the workplace’. A similar topic to Topic A has previously been found to successfully elicit a switch from the parent to the feminist identity (Zinn et al., 2023). We chose different topics related to feminist concerns to avoid effects from participants trying to recall their answers to the first writing task in the second part of the study.

#### Implicit salience measure

A pre‐trained parent‐feminist ASIA classifier (Koschate et al., 2021) was used for obtaining an implicit salience measure by determining the relative probability of a feminist identity versus a parent identity. To analyse writing style with ASIA, the Linguistic Inquiry and Word Count (LIWC) software (Pennebaker et al., 2007) was used to convert the four texts written by participants into normalized frequencies of 41 stylistic indicators. As recommended by Koschate et al. (2021), we used domain adaptation (DA; e.g. Fernando et al., 2013) to account for differences in the distribution of the online data that the classifier was originally trained on data collected in our experiment. It adjusted the classifier based on a combination of the initial forum posts used by Koschate et al. (2021) and the text provided as part of the three things manipulation writing task in the present experiment, as this task provides us with some ‘ground truth’ as to which identity should be salient. The adjusted classifier was then applied to text that participants wrote in response to the manipulation as well as to the feminist topics. The classifier provides the probability that a feminist rather than a parent identity was salient when the text was written, with 0 (*= highest probability that a parent rather than feminist identity is salient*) and 1 (= *highest probability that a feminist rather than parent identity is salient*), with 0.5 showing that the text could not be classified into either category.

#### Explicit salience measure

Directly after each writing task (for the three things manipulation and for the feminist topics), we assessed self‐reported salience. Participants answered the self‐report item ‘I am thinking of myself as a feminist[/parent] right now’ (based on Verkuyten & Hagendoorn, 1998) on a 7‐point Likert scale ranging from 1 (= *strongly disagree*) to 7 (= *strongly agree*). The identity that was asked about only related to the identity that had been manipulated as we did not wish to inadvertently make the other identity salient.

#### Demographic questionnaire

After the first survey, participants completed a demographic questionnaire, which included questions about their age, first language, gender, nationality, ethnicity, SES (Adler et al., 2000), number of children, age of oldest child and whether they currently have children living with them.

#### Task and identity questionnaire

At the end of the second survey, participants completed a task and identity questionnaire, which included items about the specific tasks in the two surveys and the identities included. It was administered after the second survey to ensure that these questions did not influence participants' responses throughout the study. First, participants were asked questions about the two writing topics: ‘The topic [“Women are still treated like sex objects”/“Women are still treated as the ‘weak gender’ in the workplace”] is typical for:’ on a 7‐point Likert scale from 1 (= *parent identity*) to 7 (= *feminist identity*). This item allowed us to determine whether there are differences in the extent to which the two topics relate to the feminist identity. The questionnaire also included exploratory items that assessed the strength of identification with each identity (based on Doosje et al., 1995; Haslam et al., 1999) and one item assessing the compatibility of the two identities (based on Benet‐Martínez & Haritatos, 2005).^2^

## RESULTS

IBM SPSS Statistics (IBM Corp, 2021) was used for all analyses, including the calculation of Bayes factors (*BF*). Noninformative priors were selected for the Bayesian analysis due to this study focusing on types of identities for which the effectiveness of identity switching has not been investigated before. We report the Bayes factor as BF_01_ (testing for evidence in favour of the null hypothesis) with scores larger than 1 being considered evidence for the null hypothesis and scores below 1 as evidence for the alternative hypothesis (for interpretation of scores, please see Lee & Wagenmakers, 2014). For each hypothesis, we report both conventional statistical analyses and the BF. We do not interpret one of these approaches to hold more weight than the other but do interpret the more conservative Bayesian analysis as providing more nuance for interpretation. Further, we included Bayesian analysis as it allows us to evaluate evidence in favour of a null hypothesis, whereas conventional statistical analyses are designed to either reject or not reject the null.

### Preliminary analyses

#### Manipulation check

**FIGURE 2:**
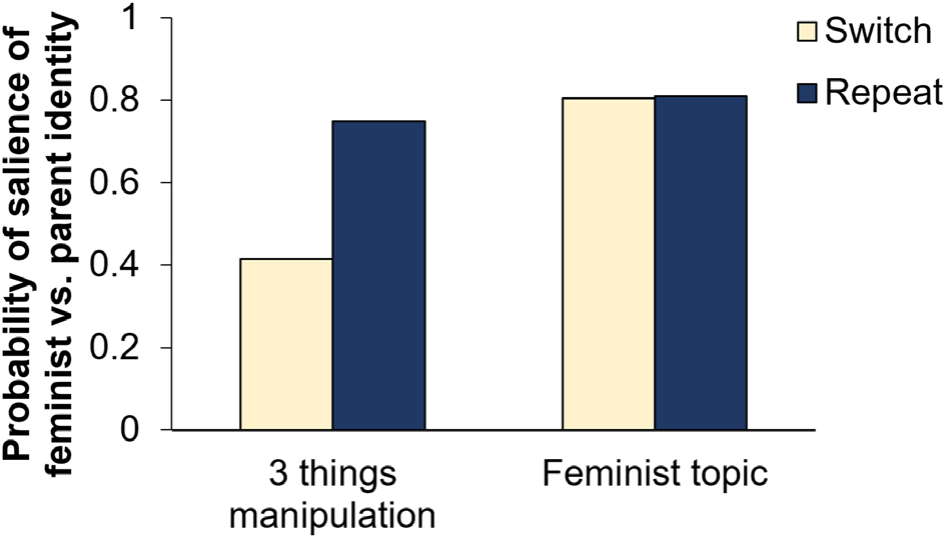
Salience of feminist vs. parent identity by condition—Study 1. A score of 0 indicates that a parent identity rather than a feminist identity is salient, a score of 0.5 indicates that it is not possible to classify the text one way or the other, and a score of 1 indicates that a feminist identity rather than a parent identity is salient.

Prior to testing the main hypotheses, we ran a manipulation check on whether the three things manipulation activated the intended identities. Participants' linguistic style differed significantly between the two conditions in the text written as part of the three things manipulation (*t*(193) = 11.02, *p* < .001, *d* = 0.79). As shown in Figure 2, participants showed a more feminist than parent linguistic style when writing about their feminist identity and a more parent than feminist linguistic style when writing about their parent identity (*M*_Diff_ = 0.33, *SE* = 0.03). Similarly, the explicit salience measure directly after the manipulation showed scores above the mid‐point of the scale for the manipulated social identity (feminist identity manipulation: *M* = 5.89, *SD* = 1.11; *t*(210) = 24.66, *p* < .001, *d* = 1.70; parent identity manipulation: *M* = 6.71, *SD* = 0.54; *t*(210) = 72.47, *p* < .001, *d* = 4.99), suggesting that social identities have been made salient as intended and that participants are to at least some extent aware of this.

#### Feminist topics

Participants rated both feminist writing topics as more typical for feminists than parents with scores significantly above the mid‐point of the scale for both topics (Topic A: *M* = 5.64, *SD* = 1.38, *t*(210) = 17.35, *p* < .001, *d* = 1.19; Topic B: *M* = 5.28, *SD* = 1.57, *t*(209) = 11.86, *p* < .001, *d* = 0.82). A paired samples *t*‐test showed that participants more strongly associated writing topic A ‘Women are still treated like sex objects’ with the feminist identity compared to writing topic B ‘Women are still treated as the “weak gender” in the workplace’ (*M*_Diff_ = 0.36, *SE* = 0.11; *t*(209) = 3.32, *p* = .001, *d* = 0.23). However, the feminist writing topics were counterbalanced across conditions and time points, and further analyses showed no influence of writing topic (Appendix B).

### Hypothesis 1: Implicit salience measure

We conducted a paired‐samples *t*‐test comparing the linguistic style in the texts written in response to the feminist topics between the switch and repeat condition to test for social identity activation costs. Consistent with H1 (the null hypothesis), no significant difference was found in the probability of the feminist versus parent identity being salient (*t*(210) = 0.24, *p* = .812, *d* = 0.02) between the switch and repeat condition (*M*_Diff_ = 0.01, *SE* = 0.02). Average probabilities indicated a feminist writing style in response to the feminist topic, regardless of whether a switch or repeat of the feminist identity had occurred. Bayesian analysis revealed strong evidence for the null hypothesis, *BF*_*01*_ = 17.78 (see ‘Main text’ panel in Figure 2 3). Since participants started with different social identities being salient in the switch vs. repeat condition (see ‘3 things manipulation’ panel in Figure 2), this indicates that participants switched from their parent to their feminist identity without incurring social identity activation costs.

We ran an exploratory repeated measures ANOVA to test for an interaction between time (three things manipulation vs. feminist topic) and condition (repeat vs. switch) on the implicit salience measure (see Table 1). We found significant main effects of time and condition as well as a significant interaction between time and condition, reflecting a significant difference in the relative salience of the feminist versus parent identities after the three things manipulation (*M*_Diff_ = 0.41, *SE* = 0.03) but a nonsignificant difference for the feminist topic (*M*_Diff_ = 0.07, *SE* = 0.03). In line with the expectation that social identity switches are effective, this suggests little persistence (inertia) in the activation of the parent identity after the switch to the feminist identity.

**TABLE 1:** 2 (time) × 2 (condition) ANOVA results—Study 1.

Effect	*F*	*df*	*p*	$\eta_{p}^{2}$
Time	144.33	1, 208	<.001	.410
Condition	86.61	1, 208	<.001	.294
Time × Condition	91.83	1, 208	<.001	.306

### Hypothesis 2: Explicit salience measure

**FIGURE 3:**
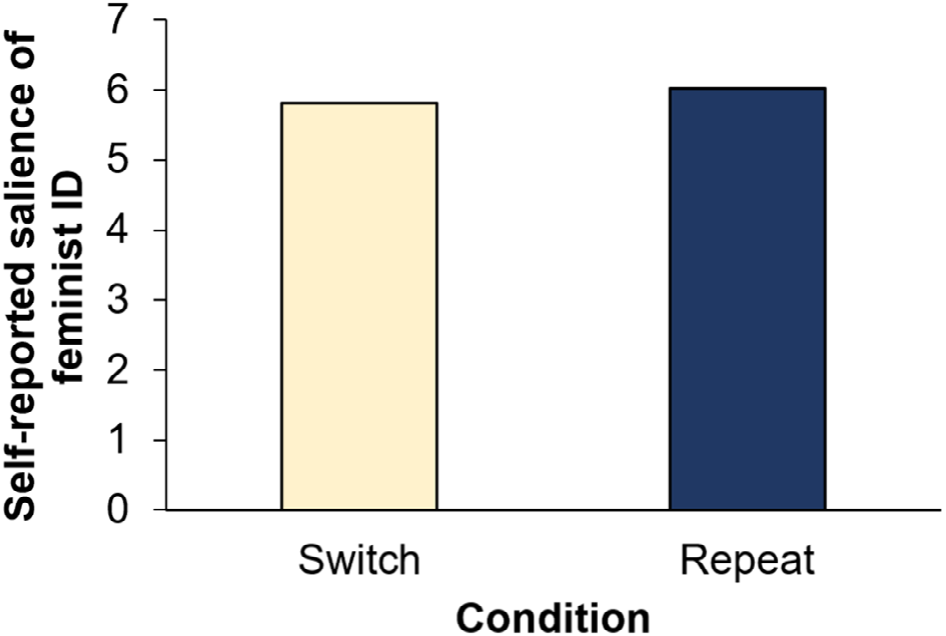
Self‐reported salience of feminist identity by condition—Study 1.

A paired‐samples *t*‐test was run to compare the two conditions on the self‐report item after the second identity (i.e. feminist identity in both conditions) had been made salient. Contrary to H2 and the finding for the implicit measure, a significant difference in the self‐reported salience scores between the switch and repeat conditions was obtained, with *t*(210) = 2.43, *p* = .016, *d* = 0.17. In line with the alternative hypothesis, the self‐reported salience scores for the feminist identity were significantly higher in the repeat than in the switch condition (*M*_Diff_ = 0.20, *SE* = 0.08; see Figure 3). However, this relatively small effect was not upheld by the Bayesian analysis, with anecdotal evidence for the null hypothesis, *BF*_*01*_ = 1.01.

### Exploratory analyses^4^

#### Change in self‐reported salience score

A comparison of the self‐report item for the feminist condition after the three things task (first identity manipulation) and the feminist writing topic (second identity manipulation) is possible for the repeat condition. A paired‐samples *t*‐test showed a significant increase in self‐reported salience scores for the feminist identity, with *t*(210) = 3.33, *p* = .001, *d* = 0.23 (*M*_first_ = 5.89, *M*_second_ = 6.02, *M*_Diff_ = 0.13, *SE* = 0.04). This indicates that the significant difference between the switch and repeat condition (as found when testing H2) is not entirely driven by a reduced salience of the feminist identity in the switch condition, as would be expected for identity activation costs. Instead, it is at least partially driven by an increase in salience of the feminist identity in the repeat condition.

## STUDY 2

In Study 2, we aim to replicate the findings with a more diverse sample and to extend the measures from the subjective experience of identity salience to also include a subjective reflection of one's own performance in a task and the difficulty of a task and of moving between tasks and identities.

### Participants and design

We aimed to recruit at least 215 participants (see Study 1 sample size calculations). We recruited participants on Prolific Academic who were aged 18 or above, were biological or adoptive parents of at least one child, fluent in English, had not taken part in Study 1 or any other social identity switching studies conducted by us and had completed at least five previous studies on Prolific. A total of 289 participants completed part 1 of the study. Of these participants, 38 (13%) were not invited to the second part because they had (1) written fewer than 25 words in at least one of the two writing tasks (*n* = 28, 10%), (2) answered that they do not support equal gender opportunity and would not consider themselves feminists (*n* = 7, 2%) or (3) submitted texts with severe spelling/grammar errors (*n* = 3, 1%). Out of these 251 participants, 239 (95%) completed the second part of the study. Of these, 29 (12%) participants were excluded from the final analyses due to insufficient word count (<25 words) in at least one of the two writing tasks in the second part of the study, and two further participants (0.8%) were excluded due to incomplete data.

Of the final *N* = 220 participants, 150 identified as women (68.2%), 69 as men (31.4%; 0.5% selected ‘other’). The average age of participants was 37.71 years (*SD* = 10.25, Min = 18, Max = 72). We widened the inclusion criteria from Study 1 to include non‐native English speakers, while ensuring that all participants reported being fluent in English. Thus, 176 participants (80%) were native English speakers (of which 69 participants were bi‐ or multi‐lingual); 41 participants (18.7%) were non‐native English speakers [of which five were bi‐ or multi‐lingual; missing responses: 3 (1.4%)]. Study 2 included participants of 27 nationalities, with the majority from South Africa (46.4%) and the United Kingdom (18.6%). Most participants indicated their ethnicity as either Black, Caribbean or African (46.8%) or White (43.2%), but the sample also included Asian or East Asian participants (4.1%) and those with Mixed or Multiple ethnicity (3.2%) as well as other or those who prefer not to say (2.8%; see Appendix C for a full list of nationalities and additional demographics).

The pre‐registered study follows the same within‐subject design as Study 1. The independent variable was identity switching (identity switch vs. repeat). The dependent variables were social identity salience (implicit ASIA measure and explicit self‐report measure) and—in addition to the variables from Study 1—the self‐reported performance in the writing task and its reported difficulty. The four balancing conditions and writing topics were the same as in Study 1. In Study 2, 56 participants started with the switch condition and Topic A (‘Women are still treated like sex objects’); 53 with the switch condition and Topic B (‘Women are still treated as the “weak gender” in the workplace’.), 57 with the repeat condition and Topic A, 54 with the repeat condition and Topic B.^5^ The study received ethics approval from the University of Exeter departmental ethics committee.

### Materials and procedure

The study followed the same procedure as Study 1. Participants could only complete the survey on a computer (to avoid sentence completion and pop‐up messages), and we also disabled copy‐pasting of text into Qualtrics to prevent the use of AI‐generated text. We used the same materials as in Study 1 with the following additions and alterations:

#### Implicit salience measure

We used the same parent‐feminist ASIA classifier (Koschate et al., 2021) as in Study 1 that had undergone domain adaptation based on text from the three things task of Study 1. Hence, the ASIA classifier was not trained or adapted based on Study 2 data; it was identical to the classifier used in Study 1.

#### Explicit salience measure

In Study 2, participants were asked about the salience of both their parent and feminist identity (in randomized order) using the same item as in Study 1, but only after the main writing task. This change relative to Study 1, where after the main writing task participants self‐reported only the salience of the feminist identity, allowed us to obtain a self‐reported salience score for each identity in each condition without affecting the salience of identities before the main writing task.

#### Self‐reported performance and difficulty measures

In Study 2, we also included items to assess how participants rated their own performance in the writing task and the self‐reported difficulty. Participants completed these measures after the main writing task. They saw the following instructions: ‘Please answer a few questions about the second writing task you completed. The topic was: [display topic A/B]’. We then asked participants to rate how compelling they found their own text (1 = *not compelling at all* to 7 = *very compelling*) and how good the quality of their text was (1 = *far below average* to 5 = *far above average*). These two items were combined into a performance scale (*r* = .58, *p* < .001). We assessed the difficulty of the writing task with three items (Example item: ‘The topic was easy to write about’). Excluding the item ‘I felt distracted during the writing process’ led to a higher scale reliability. We, therefore, formed a two‐item difficulty scale (*r* = .66, *p* < .001). We further assessed the difficulty of moving between the writing topics (‘It was easy for me to move on from the first writing task to the second writing task’) and between the two identities (‘It was challenging to move from thinking of myself as a parent to this writing task’ (reverse scored)) on a scale from 1 = *disagree completely* to 7 = *agree completely*.^6^

## RESULTS

### Preliminary analyses

#### Manipulation check

**FIGURE 4:**
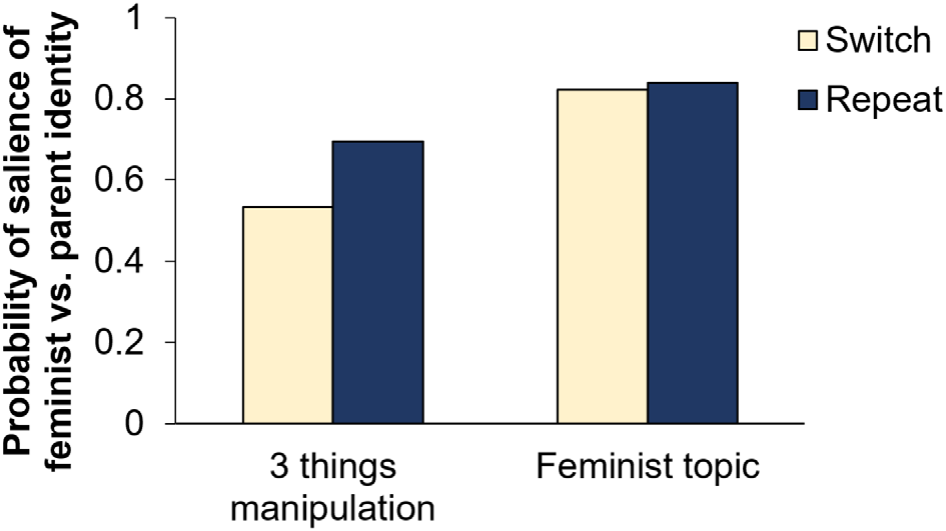
Salience of feminist vs. parent identity by condition—Study 2. A score of 0 indicates that a parent identity rather than a feminist identity is salient, a score of 0.5 indicates that it is not possible to classify the text one way or the other, and a score of 1 indicates that a feminist identity rather than parent identity is salient.

We ran a manipulation check to test whether the three things manipulation indeed activated the target social identities. The linguistic style of participants differed significantly (*t*(219) = 5.10, *p* < .001, *d* = 0.34; see also Figure 4) with a more feminist than parent linguistic style after the three things manipulation intended to activate the feminist identity and a comparatively more parent linguistic style after the manipulation intended to activate the parent identity (*M*_Diff_ = 0.16, *SE* = 0.03).

#### Feminist topics

As in Study 1, both writing topics were rated as overall more typical for feminists than for parents with scores significantly higher than the mid‐point of the scale (Topic A: *M* = 5.58, *SD* = 1.42, *t*(218) = 16.48, *p* < .001, *d* = 1.11; Topic B: *M* = 5.32, *SD* = 1.55, *t*(218) = 12.62, *p* < .001, *d* = 0.85) with topic A ‘Women are still treated like sex objects’ being associated more strongly with the feminist identity than writing topic B ‘Women are still treated as the “weak gender” in the workplace’ (*M*_Diff_ = 0.26, *SE* = 0.12; *t*(218) = 2.23, *p* = .027, *d* = 0.15). We counterbalanced, as in Study 1, the writing topics over conditions and time points. Further analyses showed no interaction between the first writing topic and condition (Appendix B).

### Hypothesis 1: Implicit salience measure

We found no significant difference in the implicit salience measure (*t*(219) = 0.81, *p* = .418, *d* = 0.06) between the switch and repeat condition (*M*_Diff_ = 0.02, *SE* = 0.02). This finding is consistent with H1, which states that there are no identity activation costs after a social identity switch. Bayesian analysis supported this finding with strong evidence for the null hypothesis (*BF*_01_ = 13.47). As shown in Figure 4, participants started with different identities being salient after the manipulation of the first identity but displayed a more feminist than parent writing style in the main writing task, irrespective of whether they had started with a feminist (repeat condition) or parent identity (switch condition).

An exploratory repeated measures ANOVA revealed significant main effects of time (3 things manipulation vs. feminist writing task; *F*(1,219) = 123.73, *p* < .001, $\eta_{p}^{2}$ = .361) and condition (switch vs. repeat; *F*(1,219) = 21.53, *p* < .001, $\eta_{p}^{2}$ = .090) as well as a significant interaction effect (*F*(1,219) = 15.78, *p* < .001, $\eta_{p}^{2}$ = .067). The difference in salience between the conditions was higher after the three things task (*M*_Diff_ = −0.16, *SE* = 0.03) than after the main writing task (*M*_Diff_ = −0.02, *SE* = 0.02) in line with expectations, supporting an effective social identity switch (H1).^7^

### Hypothesis 2: Explicit salience measure

**FIGURE 5:**
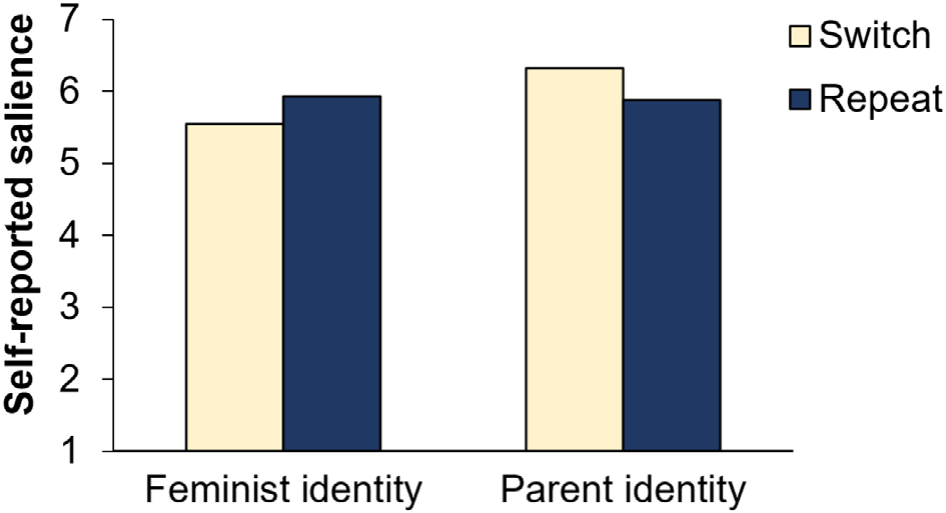
Self‐reported salience of feminist identity by condition.

Based on the updated explicit salience measure, we compared both the self‐reported salience of the parent identity and that of the feminist identity after the main writing task between the switch and repeat condition (see Figure 5). Both salience measures differed significantly between the two conditions: Participants reported significantly higher salience scores for the feminist identity in the repeat as compared to the switch condition (*M*_Diff_ = 0.40, *SE* = 0.09, *t*(219) = 4.19, *p* < .001, *d* = 0.28) while the salience scores for the parent identity were significantly lower in the repeat compared to the switch condition (*M*_Diff_ = −0.44, *SE* = 0.12, *t*(219) = −3.66, *p* < .001, *d* = 0.25). These results do not support our null hypothesis (H2) but are instead in line with perceived identity activation costs: participants perceived higher levels of parent identity salience and lower levels of feminist identity salience after the feminist writing task when they had switched from a parent identity to a feminist identity compared with a repetition of the feminist identity. In line with this, Bayesian analyses did not support the null hypothesis for the parent identity salience scores (*BF*_01_ = 0.03) and the feminist identity salience scores (*BF*_01_ = 0.004).

### Hypothesis 3: Performance ratings

To test H3, we conducted paired‐samples t‐tests to examine whether participants' own performance ratings of their texts about two feminist topics differed depending on whether they had written the text after a social identity switch or repeat. We found no significant differences between conditions in scores on the performance scale (*M*_repeat_ = 4.67, *M*_switch_ = 4.70, *M*_Diff_ = −0.04, *SE* = 0.05, *t*(219) = −0.85, *p* = .394, *d* = 0.06, *BF*_01 =_ 13.00), supporting our null hypothesis.

### Hypothesis 4: Difficulty ratings

To test H4 that the task of writing about a feminist topic will be equally easy/difficult after a social identity switch as after a social identity repeat, we conducted a further set of paired samples *t*‐tests. As Table 2 shows, the null hypothesis was supported for ratings of the difficulty of the writing task. Further, the two conditions showed no significant differences in how distracted participants felt and in the self‐reported difficulty to move between the identities. This was further in line with Bayesian analysis, which showed strong evidence for the null hypothesis for these variables. Participants did perceive the move from the first to the second writing topic as slightly easier in the repeat compared to the switch condition (*M*_repeat_ = 5.65, *M*_switch_ = 5.44, *M*_Diff_ = 0.21, *SE* = 0.10). However, Bayesian analysis did not support the robustness of this small effect with anecdotal evidence for the null hypothesis.

**TABLE 2:** Paired sample *t*‐test and Bayesian analyses for difficulty items.

Items	*t*	*df*	*p*	*d*	*BF*_01_
Writing task difficulty (scale)	−0.68	218	.498	.046	14.82
Feeling distracted	0.57	219	.571	.038	15.91
Difficulty to move between writing tasks	2.02	219	.045	.136	2.52
Difficulty to move between identities	−0.95	219	.344	.064	11.95

## DISCUSSION

Recent studies have found that people can switch social identities very effectively (Zinn et al., 2022). In the current studies, we aimed to (1) test the effectiveness of identity switching for identities which are non‐demographic and which are not distinguishable based on physical appearance, (2) use a new implicit measure of social identity salience to obtain converging evidence on the presence or absence of identity activation costs compared with an IAT assessment and (3) assess the experience of identity switches via self‐reports and performance ratings.

To address the first two aims, we tested for identity activation costs in the switch between two non‐demographic identities using ASIA as an identity salience measure (Koschate et al., 2021). We compared the implicit salience scores when participants switched between their parent and feminist identity vs. when they remained in their feminist identity. Across both studies, conventional statistical analyses revealed no significant difference in identity activation between the salience scores of the switched‐to feminist identity vs. the repeated feminist identity. Bayesian analyses provided strong support for the null hypothesis that there were no identity activation costs associated with an identity switch. These findings are in line with the previous study based on IAT‐derived measures of salience for demographic groups (Zinn et al., 2022). This supports the conclusion that social identity switches are very effective and activation cost‐free.

With regard to our first aim, we extend the previous findings based on demographic social identities, which tend to have distinct physical appearances (Zinn et al., 2022), to social identities that are not based on demographic groups and which cannot be distinguished perceptually. We show that social identity switches are also effective for the latter identities. Continuing to examine social identity switching for different types of social identities promises important practical benefits as people need to navigate between different social identities on a daily basis. Similar to the identity switches triggered in our studies, people are likely to make multiple identity switches even when the environment/context remains the same, for example, when they write a social media post to debate a societal cause on their phone while looking after their children. In line with self‐categorisation theory (Turner et al., 1987), our findings suggest that a simple task (e.g. writing a brief opinion on a topic) can rapidly make the task‐relevant social identity salient, resulting in an effective/switch from one salient identity to another. It must be said, however, that in our paradigm, participants did not experience distracting stimuli associated with other identities, as parents do when they perform tasks in the presence of their children. Hence, future research will have to examine how effective the switches are in the face of such distractions by environmental stimuli.

With regard to our second aim of examining a different method to study social identity switching, we have extended the application of ASIA from a recent study that examined whether participants can intentionally prevent an identity switch (Zinn et al., 2023) to the comparison of a social identity switch vs. repeat. In contrast to the IAT‐based switching paradigm, ASIA can be applied more easily to social identities that are indistinguishable based on perceptual features. Hence, computational (machine learning) procedures such as natural language processing (NLP) tools are promising for research on social identity switching. In particular, by relying on a relatively brief writing task, ASIA can increase the temporal resolution (precision) of measuring identity salience, while also considerably reducing the duration of the key experimental manipulation—the latter can reduce the length of the experiment and/or increase the number of observations and/or conditions during the experiment. However, one characteristic of classifier‐based procedures such as ASIA is that the relevant classifier has to be trained and validated prior to the experiment on large amounts of data and then tested and validated first. This limits the range of social identities to which ASIA can be applied. That said, previously used measures such as the IAT would also require validation of selected stimuli to ensure they are in line with the social identities of interest.

A limitation of our study is that we only included participants who speak English fluently. However, opening our Study from native English speakers (Study 1) to fluent speakers of English (Study 2) allowed us to recruit a more diverse sample, particularly with regards to ethnicity, with roughly equal numbers of White and Black participants in Study 2, and substantial numbers of participants from North America, Europe and Africa. Notably, Study 2 replicated findings from Study 1, even though the particular concerns of being a parent and achieving equal gender opportunity are likely to differ widely between ethnicities, cultures and nations. Similarly, the studies included substantial numbers of men and women who identified as both a parent and a feminist/person who supports equal gender opportunities. Our findings speak to the notion that the salience of a widely shared social identity activates core norms and values (Cork et al., 2023) that transcend demographic boundaries and specific concerns and which form the basis of ASIA as a toolkit (Koschate et al., 2021).

Our third aim was to obtain self‐report measures of the experience of identity switching in addition to the implicit measure. As with the implicit measure, we tested the null hypothesis that there is no significant difference between the self‐reported salience during an identity switch vs. repeat. In Study 1, we unexpectedly found that participants reported a higher salience of the feminist identity after the main writing task in the repeat condition than in the switch condition. However, we viewed those results with caution as the effect was small, and the more conservative Bayesian analysis showed anecdotal evidence for the null, which indicates that the effect is not likely to be robust. Further, we found that this difference was at least partly driven by an increase in feminist identity salience in the repeat condition, which would not be consistent with identity activation cost in the switch condition. This might have been caused by how the explicit measure was collected. In Study 1, participants in the repeat condition were asked about the salience of their feminist identity after the three things task and then again after the main writing task. This repetition of the question might have led participants in the repeat condition to answer that they indeed thought ‘more’—in the sense of ‘longer’ —about themselves as a feminist. To address these limitations and further assess whether social identity switching might lead to perceived identity activation costs, we only assessed explicit salience once (after the main writing task) in Study 2. In Study 2, we did indeed find further support for perceived identity activation costs. Nevertheless, even the changes in Study 2 cannot fully mitigate against some of the key limitations of self‐report salience items. Specifically, the potential lack of introspection into which identity is salient and the potential that the item itself might have caused a switch in salience. Therefore, more research will be needed to examine which costs people might be experiencing even though implicitly it appears that they did switch effectively. We took a first step in answering this question by focusing on potential effects on people's self‐reported performance.

In Study 2, we also measured participants' self‐reported performance in the main writing task, the reported difficulty of the writing task, and of moving from the first to the second writing task. We added this measure to learn more about potential perceived switch costs. However, we found little‐to‐no difference in these measures between the switch and control conditions. The only item for which we found a weak difference (not supported by Bayesian analyses) asked participants how easy they found it to move from the first to the second writing task. Participants reported this as significantly more difficult when they completed the switch condition compared with the repeat condition. However, it is interesting to note that the only significant difference was found for an item that referred to a change in task rather than identity. This may point towards a perception of task (theme) switch costs, that is, a perception that a move from writing about being a parent to writing about a feminist topic is a bigger task switch compared with a move from writing about being a feminist to a feminist topic. However, future research is needed to determine with more certainty whether participants might experience effects of an identity switch on self‐reported, and even actual, performance and the difficulty of the task. In particular, it would be important to understand whether multiple switches in quick succession may affect performance, whether individual differences exist, and the extent to which switching identities affects other outcomes, such as subjective well‐being.

Among avenues for future research, we would envisage investigating switching between conflicting or even incompatible social identities and between negatively valued identities. Present and previous studies of social identity switching focused on positively or neutrally valenced identities (Zinn et al., 2022, 2023). Stigmatized groups defined as a separate identity cluster by Deaux et al. (1995; see also Cork et al., 2023) should constitute an important subject for future research into social identity switching. Similarly, understanding whether individuals with high levels of identity complexity (Roccas & Brewer, 2002) navigate switches differently from those with low levels of identity complexity may provide important insights into the cognitive underpinnings of social identities.

## CONCLUSION

The current studies directly compared switches between non‐demographic identities with repetitions of such identities using (1) a novel computational‐linguistic technique (ASIA) that allows for implicit measurement of the relative salience of social identities and (2) explicit measures based on self‐report. As expected, we found no identity activation costs after a social identity switch when assessed with an implicit measure and also found no effect of switching on the subjective ratings of own performance or task difficulty. In contrast, explicit measures of identity salience and difficulty of moving between tasks suggested an effect of social identity switching in line with switch costs, albeit with only weak evidence. We conclude that switching between non‐demographic identities is highly effective despite participants' somewhat contrary indications through self‐reporting. These findings open up interesting avenues for future research on other non‐demographic social identities, including stigmatized identities, and on whether there are other perceived costs associated with the highly effective cognitive process of switching social identities.

## AUTHOR CONTRIBUTIONS

**Anna Kristina Zinn:** Conceptualization; data curation; formal analysis; funding acquisition; investigation; methodology; project administration; software; supervision; validation; visualization; writing – original draft; writing – review and editing. **Aureliu Lavric:** Conceptualization; funding acquisition; methodology; supervision; validation; writing – review and editing. **Elahe Naserianhanzaei:** Methodology; software. **Miriam Koschate:** Conceptualization; data curation; formal analysis; funding acquisition; investigation; methodology; software; supervision; validation; writing – review and editing.

## CONFLICT OF INTEREST STATEMENT

The authors declare no conflicts of interest.

## Data Availability

The data that support the findings of this study are openly available upon OSF: https://osf.io/sfvjt.

## Footnotes

^1^
The conditions were balanced slightly imperfectly due to exclusions.

^2^
These items were included for exploratory purposes only and will therefore not be reported in the results section.

^3^
Since the present studies have within‐subject designs, the SEs of individual means are not informative regarding the variability of the contrasts of interest (e.g., the difference between congruent and incongruent trials). Therefore, they are not provided in the text or in the figures. We report, however, the SEs of the contrasts.

^4^
Additional exploratory analyses can be found in Appendix D.

^5^
The conditions were balanced slightly imperfectly due to exclusions.

^6^
We further included an additional exploratory item assessing emotions which did not form part of the main analyses.

^7^
Additional exploratory analyses can be found in Appendix D.

## APPENDIX A ADDITIONAL DEMOGRAPHICS: STUDY 1

Participants rated their socio‐economic status (SES) at *M* = 5.51 (*SD* = 1.76, min = 0, max = 10) on a scale from 0 (= worst off in society) to 10 (= best off in society). They had, on average, two children (*SD* = 0.86, min = 1, max = 6), with the youngest child being on average 10.22 years old (*SD* = 8.53, min = 0, max = 39); *n* = 191 participants (90.5%) indicated that they currently had children living in their household. The nationalities of participants are summarised in Table A1.

**TABLE A1:** Participant's nationalities: Study 1.

Nationality	Frequency	Percentage
United Kingdom	121	57.3
South Africa	26	12.3
United States of America	24	11.4
Canada	9	4.3
Ireland	6	2.8
Australia	4	1.9
Zimbabwe	4	1.9
New Zealand	3	1.4
India	1	0.5
Italy	1	0.5
Jamaica	1	0.5
Sweden	1	0.5
Missing information	10	4.7

## APPENDIX B ADDITIONAL ANALYSES FOR ORDER OF WRITING TOPICS

### STUDY 1

We found no significant interaction between the order of writing topic and the repeat vs. switch condition on the linguistic style salience measure (*F*(1, 207) = 0.74, *p* = .391, $\eta_{p}^{2}$ = .004). Further, there was no main effect of the order of writing topic on the implicit salience measure scores overall (*M*_Diff_ = 0.001, *SE* = 0.04; *F*(1, 207) = 0.001, *p* = .979, $\eta_{p}^{2}$ < .001). We, therefore, analysed the data aggregated over the two topics.

### STUDY 2

As in Study 1, we found no significant interaction between the order of writing topics and the repeat vs. switch condition on the implicit salience measure (*F*(1, 218) = 1.07, *p* = .302, $\eta_{p}^{2}$ = .005). However, it should be noted that we did find an overall effect of the order of writing topic on the linguistic style salience measure (*F*(1, 218) = 8.16, *p* = .005, $\eta_{p}^{2}$ = .036), with a stronger feminist linguistic style if participants started with topic A compared to topic B (*M*_topicA_ = 0.86; *M*_topicB_ = 0.80; *M*_Diff_ = 0.07, *SE* = 0.02). However, since this did not affect the two conditions (switch and repeat) differently, we proceeded with our main analyses as pre‐registered (analysing the data aggregated over the two topics).

## APPENDIX C ADDITIONAL DEMOGRAPHICS: STUDY 2

Participants on average scored their SES as *M* = 5.81 (*SD* = 1.60, min = 0, max = 10) and had on average two children (*SD* = 0.93, min = 1, max = 7). Their youngest child was on average 8 years old (*SD* = 7.66, min = 0, max = 41), and 204 participants (92.7%) currently have children living in their household. The nationalities of participants are summarised in Table A2.

**TABLE A2:** Participant's nationalities: Study 2.

Nationality	Frequency	Percentage
South Africa	102	46.4
United Kingdom	41	18.6
Zimbabwe	9	4.1
Portugal	8	3.6
Ireland	6	2.7
Italy	5	2.3
Poland	5	2.3
Hungary	4	1.8
Spain	4	1.8
Australia	3	1.4
Greece	3	1.4
Nigeria	3	1.4
Brazil	2	0.9
Netherlands	2	0.9
Slovenia	2	0.9
Sweden	2	0.9
Turkey	2	0.9
Czech Republic	1	0.5
Denmark	1	0.5
Estonia	1	0.5
Germany	1	0.5
India	1	0.5
Lithuania	1	0.5
New Zealand	1	0.5
Russian Federation	1	0.5
Kingdom of Eswatini	1	0.5
United States of America	1	0.5
Missing information	7	3.2

## APPENDIX D ADDITIONAL EXPLORATORY ANALYSES

### FAMILY‐RELATED WORDS: STUDY 1

We compared the percentage of family‐related words that participants used in the texts written about the feminist topics (i.e. the number of family words relative to their total word count) based on the LIWC feature ‘family’. The repeat and switch condition did not differ significantly in the number of family‐related words (*M*_repeat_ = 0.20; *M*_switch_ = 0.26; *M*_Diff_ = −0.06, *SE* = 0.07; *t*(210) = −0.85, *p* = .398, *d* = 0.06, *BF* = 12.80).

### TIME SPENT ON WRITING TASK: STUDY 1

Since the writing tasks had no time limit, one alternative explanation for the absence of identity activation costs in the implicit salience measure is that participants in the switch condition might have slowed down the feminist topic writing task, which may have allowed enough time to switch over to their feminist identity. To test for this explanation, a paired‐samples *t*‐test compared the time participants spent writing in the switch condition to the time spent writing in the repeat condition. The marginally significant difference between the conditions in the time taken (*t*(210) = 1.84, *p* = .068, *d* = 0.13, *BF*_01_ = 3.46) was in the opposite direction—participants spent marginally more time writing about the feminist topic in the repeat condition than in the switch condition (*M*_repeat_ = 232.29 s, *M*_switch_ = 204.12 s, *M*_Diff_ = 28.17 s, *SE* = 15.35 s).

### GENDER DIFFERENCES: STUDY 1

We tested for an effect of gender (comparing male and female participants only due to the low number of participants in other gender categories) on the main outcome variables. We used independent samples *t*‐tests (Welch's *t*‐test if equal variance could not be assumed) and corrected for multiple comparisons using Bonferroni correction. With the given number of participants in each group (male: *n* = 57; female: *n* = 153), sensitivity calculations (using GPower3.1) showed a required effect size of *d* = 0.44 for a power of .8 and an error probability of .05. Table A3 shows the effect of gender on all implicit and explicit salience measures. There was no significant difference between male and female participants on any of the implicit salience scores calculated as part of the different writing tasks. Female participants showed a significantly higher self‐reported salience of their parent identity than male participants after completing the 3 things manipulation intended to activate the parent identity. After the main writing task, female participants in both the switch and repeat conditions showed significantly higher self‐reported salience scores of the feminist identity than male participants.

**TABLE A3:** Paired sample *t*‐test for gender differences Study 1.

Items	*M* (*SD*) male	*M* (*SD*) female	*t*	*df*	*p*	*d*
Implicit salience: 3 things task (switch)	0.45 (0.38)	0.38 (0.34)	1.30	91.45	.197	0.212
Implicit salience: 3 things task (repeat)	0.76 (0.32)	0.74 (0.33)	0.48	207	.634	0.075
Implicit salience: main text (switch)	0.85 (0.19)	0.79 (0.26)	1.82	142.23	.071	0.241
Implicit salience: main text (repeat)	0.79 (0.25)	0.82 (0.23)	−0.70	208	.482	0.109
SRS: parent identity after parent 3 things task	6.49 (0.66)	6.78 (.47)	−3.08	78.48	.003*	0.554
SRS: feminist identity after feminist 3 things task	5.68 (1.26)	5.95 (1.05)	−1.57	208	.118	0.244
SRS: feminist identity after main text (switch)	5.14 (1.57)	6.06 (1.10)	−4.05	77.10	<.001*	0.739
SRS: feminist identity after main text (repeat)	5.63 (1.23)	6.16 (0.90)	−3.39	208	.001*	0.526

Abbreviation: SRS, self‐reported salience.

* Indicates values which remain statistically significant after correcting for multiple testing using Bonferroni correction (adjusted error probability level: .006); Implicit salience (Probability of salience of feminist vs. parent identity).

### FAMILY‐RELATED WORDS: STUDY 2

As in Study 1, there was no significant difference in the number of family‐related words in the main text between the repeat and switch condition (*M*_repeat_ = 0.30; *M*_switch_ = 0.33; *M*_Diff_ = −0.03, *SE* = 0.08; *t*(219) = −0.36, *p* = .717, *d* = 0.02, *BF* = 17.49).

### TIME SPENT ON WRITING TASK: STUDY 2

The repeat (*M* = 249.46 s, *SD* = 129.05 s) and switch (*M* = 267.36 s, *SD* = 188.15 s) condition showed no significant difference in the time taken to complete the main writing task (*M*_Diff_ = −17.90 s, *SE* = 11.98 s; *t*(219) = −1.49, *p* = .137, *d* = 0.10, *BF*_01_ = 6.18).

### GENDER DIFFERENCES: STUDY 2

As in Study 1, we used independent samples *t*‐tests (Welch's *t*‐test if equal variance could not be assumed) to test for an effect of gender on the main outcome variables. Sensitivity calculations with the given number of participants in each group (male: *n* = 69; female: *n* = 150) showed a required effect size of *d* = 0.41 for a power of .8 and an error probability of .05. The results are summarized in Table A4. After Bonferroni corrections, there was no significant difference between male and female participants on any of the implicit salience scores or any of the performance and difficulty measures. There was also no significant difference in the self‐reported salience scores for the parent identity. For the self‐reported salience scores of the parent identity in both the switch and repeat condition, female participants reported significantly higher scores than male participants.

**TABLE A4:** Paired sample *t*‐test for gender differences Study 2.

Items	*M* (*SD*) male	*M* (*SD*) female	*t*	*df*	*p*	*d*
Implicit salience: 3 things task (switch)	0.58 (0.36)	0.52 (0.36)	1.23	217	.219	0.179
Implicit salience: 3 things task (repeat)	0.74 (0.34)	0.67 (0.36)	1.41	217	.161	0.204
Implicit salience: main text (switch)	0.85 (0.22)	0.81 (0.25)	1.09	217	.276	0.159
Implicit salience: main text (repeat)	0.88 (0.18)	0.82 (0.23)	2.07	168.69	.040	0.274
SRS: feminist identity after main text (switch)	4.88 (1.60)	5.85 (1.24)	−4.42	106.54	<.001*	0.707
SRS: parent identity after main text (switch)	6.48 (0.82)	6.26 (1.27)	1.53	194.26	.128	0.190
SRS: feminist identity after main text (repeat)	5.41 (1.35)	6.17 (1.11)	−4.08	111.98	<.001*	0.637
SRS: parent identity after main text (repeat)	5.72 (1.54)	5.96 (1.67)	−0.99	217	.323	0.144
Self‐reported performance (switch)	4.68 (0.88)	4.72 (0.98)	−0.31	217	.760	0.044
Writing task difficulty scale (switch)	5.49 (1.39)	5.58 (1.14)	−0.49	216	.622	0.072
Feeling distracted (switch)	6.14 (1.24)	6.02 (1.33)	0.66	217	.510	0.096
Difficulty to move between topics (switch)	5.57 (1.39)	5.38 (1.55)	0.85	217	.397	0.123
Difficulty to move between identities (switch)	4.90 (1.86)	4.97 (1.85)	−0.28	217	.782	0.040
Self‐reported performance (repeat)	4.64 (0.93)	4.67 (0.92)	−0.16	217	.871	0.024
Writing task difficulty scale (repeat)	5.28 (1.29)	5.57 (1.25)	−1.55	217	.123	0.225
Feeling distracted (repeat)	6.07 (1.32)	6.14 (1.27)	−0.36	217	.718	0.053
Difficulty to move between topics (repeat)	5.42 (1.43)	5.74 (1.27)	−1.67	217	.097	0.242
Difficulty to move between identities (repeat)	4.43 (1.86)	4.94 (1.93)	−1.82	217	.070	0.265

Abbreviation: SRS, self‐reported salience.

* Indicates values that remain statistically significant after correcting for multiple testing using Bonferroni correction (adjusted error probability level: .003); Implicit salience (Probability of salience of feminist vs. parent identity).

## References

[bjso12906-bib-0001] Adler, N. E. , Epel, E. S. , Castellazzo, G. , & Ickovics, J. R. (2000). Relationship of subjective and objective social status with psychological and physiological functioning: Preliminary data in healthy, white women. Health Psychology, 19(6), 586–592. 10.1037/0278-6133.19.6.586 11129362

[bjso12906-bib-0002] Benet‐Martínez, V. , & Haritatos, J. (2005). Bicultural identity integration (BII): Components and psychosocial antecedents. Journal of Personality, 73(4), 1015–1050. 10.1111/j.1467-6494.2005.00337.x 15958143

[bjso12906-bib-0003] Brysbaert, M. (2019). How many participants do we have to include in properly powered experiments? A tutorial of power analysis with reference tables. Journal of Cognition, 2(1), 1–38. 10.5334/joc.72 31517234 PMC6640316

[bjso12906-bib-0004] Corallo, G. , Sackur, J. , Dehaene, S. , & Sigman, M. (2008). Limits on introspection: Distorted subjective time during the dual‐task bottleneck. Psychological Science, 19(11), 1110–1117. 10.1111/j.1467-9280.2008.02211.x 19076482

[bjso12906-bib-0005] Cork, A. , Everson, R. , Levine, M. , & Koschate, M. (2020). Using computational techniques to study social influence online. Group Processes & Intergroup Relations, 23(6), 808–826. 10.1177/1368430220937354

[bjso12906-bib-0006] Cork, A. , Everson, R. , Naserian, E. , Levine, M. , & Koschate, M. (2023). Collective self‐understanding: A linguistic style analysis of naturally occurring text data. Behavior Research Methods, 55, 4455–4477. 10.3758/s13428-022-02027-8 36443583 PMC9707163

[bjso12906-bib-0007] Deaux, K. , Reid, A. , Mizrahi, K. , & Ethier, K. A. (1995). Parameters of social identity. Journal of Personality and Social Psychology, 68(2), 280–291. 10.1037/0022-3514.68.2.280

[bjso12906-bib-0008] Doosje, B. , Ellemers, N. , & Spears, R. (1995). Perceived intragroup variability as a function of group status and identification. Journal of Experimental Social Psychology, 31(5), 410–436. 10.1006/jesp.1995.1018

[bjso12906-bib-0009] Fernando, B. , Habrard, A. , Sebban, M. , & Tuytelaars, T. (2013). Unsupervised visual domain adaptation using subspace alignment. In Proceedings of the IEEE international conference on computer vision, Institute of Electrical and Electronics Engineers. (pp. 2960–2967).

[bjso12906-bib-0010] Greenwald, A. , McGhee, D. , & Schwartz, J. (1998). Measuring individual differences in implicit cognition: The implicit association test. Journal of Personality and Social Psychology, 74(6), 1464–1480. 10.1037/0022-3514.74.6.1464 9654756

[bjso12906-bib-0011] Haslam, S. A. , Oakes, P. J. , Reynolds, K. J. , & Turner, J. C. (1999). Social identity salience and the emergence of stereotype consensus. Personality and Social Psychology Bulletin, 25(7), 809–818. 10.1177/0146167299025007004

[bjso12906-bib-0012] IBM Corp . (2021). IBM SPSS Statistics for Windows (Version 28.0) [Computer software]. IBM Corp.

[bjso12906-bib-0013] Kiesel, A. , Steinhauser, M. , Wendt, M. , Falkenstein, M. , Jost, K. , Philipp, A. M. , & Koch, I. (2010). Control and interference in task switching – A review. Psychological Bulletin, 136(5), 849–874. 10.1037/a0019842 20804238

[bjso12906-bib-0014] Koschate, M. , Naserian, E. , Dickens, L. , Stuart, A. , Russo, A. , & Levine, M. (2021). ASIA: Automated social identity assessment using linguistic style. Behavior Research Methods, 53(4), 1762–1781. 10.3758/s13428-020-01511-3 33575985 PMC8367904

[bjso12906-bib-0015] Lavric, A. , Mizon, G. A. , & Monsell, S. (2008). Neurophysiological signature of effective anticipatory task‐set control: A task‐switching investigation. European Journal of Neuroscience, 28(5), 1016–1029. 10.1111/j.1460-9568.2008.06372.x 18717737

[bjso12906-bib-0016] Lee, M. D. , & Wagenmakers, E. J. (2014). Bayesian cognitive modeling: A practical course. Cambridge University Press.

[bjso12906-bib-0017] Lickel, B. , Hamilton, D. L. , Wieczorkowska, G. , Lewis, A. , Sherman, S. J. , & Uhles, A. N. (2000). Varieties of groups and the perception of group entitativity. Journal of Personality and Social Psychology, 78, 223–246. 10.1037//0022-3514.78.2.223 10707331

[bjso12906-bib-0018] Meiran, N. (1996). Reconfiguration of processing mode prior to task performance. Journal of Experimental Psychology: Learning, Memory, and Cognition, 22(6), 1423–1442. 10.1037/0278-7393.22.6.1423

[bjso12906-bib-0019] Mittelstädt, V. , Miller, J. , & Kiesel, A. (2018). Trading off switch costs and stimulus availability benefits: An investigation of voluntary task‐switching behavior in a predictable dynamic multitasking environment. Memory & Cognition, 46, 699–715. 10.3758/s13421-018-0802-z 29524178

[bjso12906-bib-0020] Monsell, S. (2003). Task switching. Trends in Cognitive Sciences, 7(3), 134–140. 10.1016/S1364-6613(03)00028-7 12639695

[bjso12906-bib-0021] Monsell, S. (2015). Task‐set control and task switching. In J. Fawcett , E. F. Risko , & A. Kingstone (Eds.), The handbook of attention (pp. 139–172). MIT Press.

[bjso12906-bib-0022] Monsell, S. , & Mizon, G. (2006). Can the task‐cuing paradigm measure an endogenous task‐set reconfiguration process? Journal of Experimental Psychology: Human Perception and Performance, 32(3), 493–516. 10.1037/0096-1523.32.3.493 16822121

[bjso12906-bib-0023] Oakes, P. J. (1987). The salience of social categories. In J. C. Turner , M. A. Hogg , P. J. Oakes , S. D. Reicher , & M. S. Wetherell (Eds.), Rediscovering the social group (pp. 117–141). Basil Blackwell.

[bjso12906-bib-0024] Pennebaker, J. W. , Booth, R. J. , & Francis, M. E. (2007). Linguistic inquiry and word count: LIWC [Computer software]. liwc.net.

[bjso12906-bib-0025] Pinter, B. , & Greenwald, A. (2010). A comparison of minimal group induction procedures. Group Processes & Intergroup Relations, 14(1), 81–98. 10.1177/1368430210375251

[bjso12906-bib-0026] Prentice, D. A. , Miller, D. T. , & Lightdale, J. R. (1994). Asymmetries in attachments to groups and to their members: Distinguishing between common‐identity and common‐bond groups. Personality and Social Psychology Bulletin, 20(5), 484–493. 10.1177/0146167294205005

[bjso12906-bib-0027] Roccas, S. , & Brewer, M. B. (2002). Social identity complexity. Personality and Social Psychology Review, 6(2), 88–106. 10.1207/S15327957PSPR0602_01

[bjso12906-bib-0028] Rogers, R. , & Monsell, S. (1995). Costs of a predictible switch between simple cognitive tasks. Journal of Experimental Psychology: General, 124(2), 207–231. 10.1037/0096-3445.124.2.207

[bjso12906-bib-0029] Silver, E. R. , Chadwick, S. B. , & van Anders, S. M. (2019). Feminist identity in men: Masculinity, gender roles, and sexual approaches in feminist, non‐feminist, and unsure men. Sex Roles, 80, 277–290. 10.1007/s11199-018-0932-6

[bjso12906-bib-0030] Tajfel, H. , & Turner, J. C. (1979). An integrative theory of intergroup conflict. In W. G. Austin & S. Worchel (Eds.), The social psychology of intergroup relations (pp. 33–47). Brooks/Cole.

[bjso12906-bib-0031] Turner, J. , Oakes, P. , Haslam, S. , & McGarty, C. (1994). Self and collective: Cognition and social context. Personality and Social Psychology Bulletin, 20(5), 454–463. 10.1177/0146167294205002

[bjso12906-bib-0032] Turner, J. C. , Hogg, M. A. , Oakes, P. J. , Reicher, S. D. , & Wetherell, M. S. (1987). Rediscovering the social group: A self‐categorization theory. Blackwell.

[bjso12906-bib-0033] Verkuyten, M. , & Hagendoorn, L. (1998). Prejudice and self‐categorization: The variable role of authoritarianism and in‐group stereotypes. Personality and Social Psychology Bulletin, 24(1), 99–110. 10.1177/0146167298241008

[bjso12906-bib-0034] Zinn, A. K. , Koschate, M. , Naserianhanzaei, E. , & Lavric, A. (2023). Can we prevent social identity switches? An experimental‐computational investigation. The British Journal of Social Psychology, 62(3), 1547–1565. 10.1111/bjso.12647 37039361 PMC10947443

[bjso12906-bib-0035] Zinn, A. K. , Lavric, A. , Levine, M. , & Koschate, M. (2022). Social identity switching: How effective is it? Journal of Experimental Social Psychology, 101 Atricle 104309., 104309. 10.1016/j.jesp.2022.104309

